# Role of the Red Ginseng in Defense against the Environmental Heat Stress in Sprague Dawley Rats

**DOI:** 10.3390/molecules201119692

**Published:** 2015-11-10

**Authors:** Kui-Jin Kim, Kye-Yoon Yoon, Hee-Do Hong, Boo-Yong Lee

**Affiliations:** 1 Department of Food Science and Biotechnology, College of Life Science, CHA University, Kyonggi 463-400, Korea; Kuijin.Kim@gmail.com (K.-J.K.); beautyygy@naver.com (K.-Y.Y.); 2 Korea Food Research Institute, Kyonggi, Seongnam 463-746, Korea; honghd@kfri.re.kr

**Keywords:** red ginseng, herbal medicine, heat environmental stress, lipid peroxidation, lipid accumulation, ginsenoside

## Abstract

Global temperature change causes heat stress related disorders in humans. A constituent of red ginseng has been known the beneficial effect on the resistance to many diseases. However, the mechanism of red ginseng (RG) against heat stress still remains unclear. To determine the effect of RG on heat stress, we examined the effect of the RG on the gene expression profiles in rats subjected to environmental heat stress. We evaluated the transcripts associated with hepatic lipid accumulation and oxidative stress in rats subjected to heat stress. We also analyzed the reactive oxygen species (ROS) contents. Our results suggested RG inhibited heat stress mediated altering mRNA expressions include HSPA1, DEAF1, HMGCR, and FMO1. We also determined RG attenuated fat accumulation in the liver by altering C/EBPβ expression. RG promoted to repress the heat stress mediated hepatic cell death by inhibiting of Bcl-2 expression in rats subjected to heat stress. Moreover, RG administered group during heat stress dramatically decreased the malondialdehyde (MDA) contents and ROS associated genes compared with the control group. Thus, we suggest that RG might influence inhibitory effect on environmental heat stress induced abnormal conditions in humans.

## 1. Introduction

The global temperature has increased by approximately 0.4 °C over the last 150 years [1]. The change causes increasing environmental heat stress related disorders including infectious diseases in humans [2,3]. Physiological responses to environmental heat stress are grouped as primary, which include endocrine changes such as in measurable levels of circulating cytokines and corticosteroids [4,5], and secondary changes in gene expression including specific and highly regulated signaling cascades leading to the transcriptional regulation of endogenous antioxidant enzymes [6,7,8]. We also reported that most responsible for environmental heat stress are lipid metabolism associated transcription factors including 3-hydroxy-3-methylglutaryl CoA reductase (HMGCR), flavin containing monooxygenase 1 (FMO1), deformed epidermal auto regulatory factor 1 (DEAF1), and heat shock protein 70 (HSPA1) in rats [9]. Hence, it is important to attenuate the lipid metabolism associated transcription factors to prevent environmental stress mediated diseases.

In previous, we suggested that botanical plant extracts such as *Acanthopanax senticosus* and *Schisandra chinensis* reduced environmental heat stress caused abnormal hepatic gene expression [9,10]. The repression of the lipid metabolism associated biomarker is essential to prevent environmental heat stress mediated disease incidence. The botanical plant has been used for several centuries for a wide variety of medicinal purposes, including the balance of regulative and detoxification functions. *Panax ginseng* is a famous botanical plant and is widely used as a preventive or therapeutic herbal medicine globally. Depending on manufacturing processes, ginseng is available in three different forms: white, red, and black ginseng [11,12,13].

In particular, red ginseng (RG) is produced by steaming and drying ginseng root grown several times. RG has a lot of bioactive ingredients compared with other types of manufactured ginseng. The major active components of RG include ginsenosides (Rg1, Rg3, Rb1 and Rb2), polysaccharides, peptides, polyacetylenic alcohols, and fatty acids. 

A number of ginseng extracts provide beneficial effects have been reported in the last few years including resistance to neuronal disease, tumor progression, and inflammation [14,15,16,17,18]. These reports suggest that RG regulate oxidative stress through the inactivation of nuclear factor kappa-light-chain-enhancer of activated B (NF-κB) that promote the abolishing transcription factors of inducible nitric oxide synthase (iNOS) and cyclooxygenase 2 (COX2) and subsequently decrease the oxidative stress response in the cell [19,20]. Oxidative stress is one of the crucial factors of environmental heat stress. However, the molecular mechanism of RG on heat stress still remains unclear. Hence, we examined that the effect of the RG on the hepatic gene expression in rats subjected to environmental heat stress. 

## 2. Results

### 2.1. Extraction Process and Chemical Compositions of Red Ginseng 

**Figure 1:**
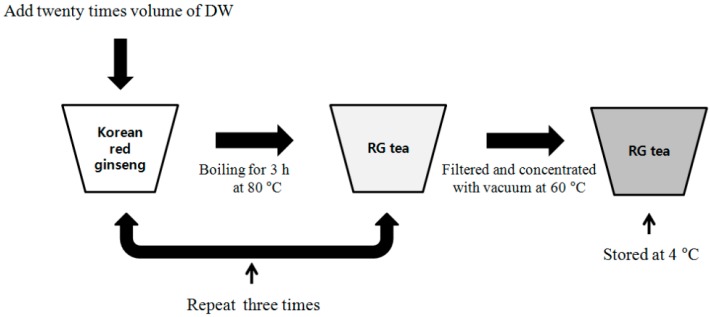
Red ginseng extracts (RG) preparation process.

RG was extracted with deionized water at 20 times volume and this process was carried out for 3 h at 80 °C and was repeated three times. Total RG extracts were filtered, concentrated under vacuum at 60 °C and stored at 4 °C until use (Figure 1). 

The extracts of RG were containing oligosaccharide (60.57 mg), acidic polysaccharide (7.58 mg), phenolic compound (0.92 mg), and saponin (5.58 mg) as shown in Table 1. The ginsenosides Rb1 and Rg1 are regarded as the major compounds responsible for many pharmaceutical actions of ginseng. Both Rb1 and Rg1 measured 0.26 mg and 0.18 mg in RG, respectively.

**Table 1:** Chemical composition of red ginseng (RG). Analyzed extracts from 100 mg Korean red ginseng.

Oligosaccharide	Acidic Polysaccharide	Phenolic Compounds	Saponin	Ginsenoside	
				Rb1	Rg1
60.57 mg	7.58 mg	0.92 mg	5.58 mg	0.26 mg	0.18 mg

### 2.2. Body Weights, Food Intake, and Water Conservation

We assessed body weight, diet, and water conservation to evaluate differences between heat stresses exposed and heat stress exposed with RG groups in SD rats.

**Figure 2:**
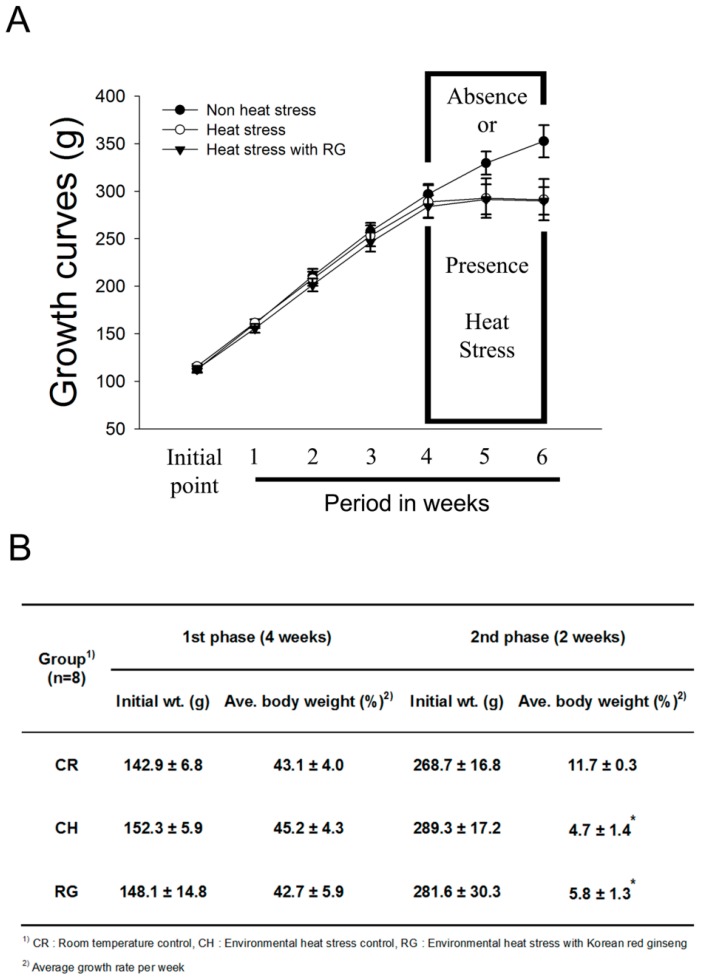
Growth rate for SD rats exposed to heat stress with and without RG for six weeks. (**A**) Effect of red ginseng on growth rates of the environmental heat stress exposed SD rats; (**B**) Mean of body weight change of SD rats housed under a room temperature for 4 weeks and with environmental heat stress with red ginseng (RG). Data represent the means ± standard deviation (*n* = 10). * *p* < 0.05 compared with room temperature control.

Body weights were measured at the initiation of treatment, every week thereafter, and on the day of sacrifice. No animals died during the experimental period. The body weight changes of all groups have no significant difference in initial periods, however, there were significantly inhibited growth ratios during in heat stress exposed (4.7%) and heat stress exposed to RG group (5.8%) compared with the control (11.7%) as shown in Figure 2A,B. They were suppressed in the organ weight in both the heat stress exposed and heat stress exposed with RG groups compared to the control group as shown in Table 2.

**Table 2:** Absolute organ weights of SD rats.

Groups (*n* = 10)	Organ Weights (g)				
	Liver	Kidney	Lung	Brain	Spleen
Non heat stress	10.08 ± 0.69	2.50 ± 0.15	1.48 ± 0.13	1.90 ± 0.04	0.75 ± 0.08
Heat stress	7.31 ± 0.87 *	2.19 ± 0.21 *	1.29 ± 0.14 *	1.86 ± 0.06	0.53 ± 0.08 *
Heat stress with RG	7.04 ± 0.47 *	2.25 ± 0.13 *	1.22 ± 0.15 *	1.92 ± 0.08	0.55 ± 0.05 *

Data represent the means ± standard deviation (*n* = 10). * *p* < 0.05 compared with room temperature control.

**Figure 3:**
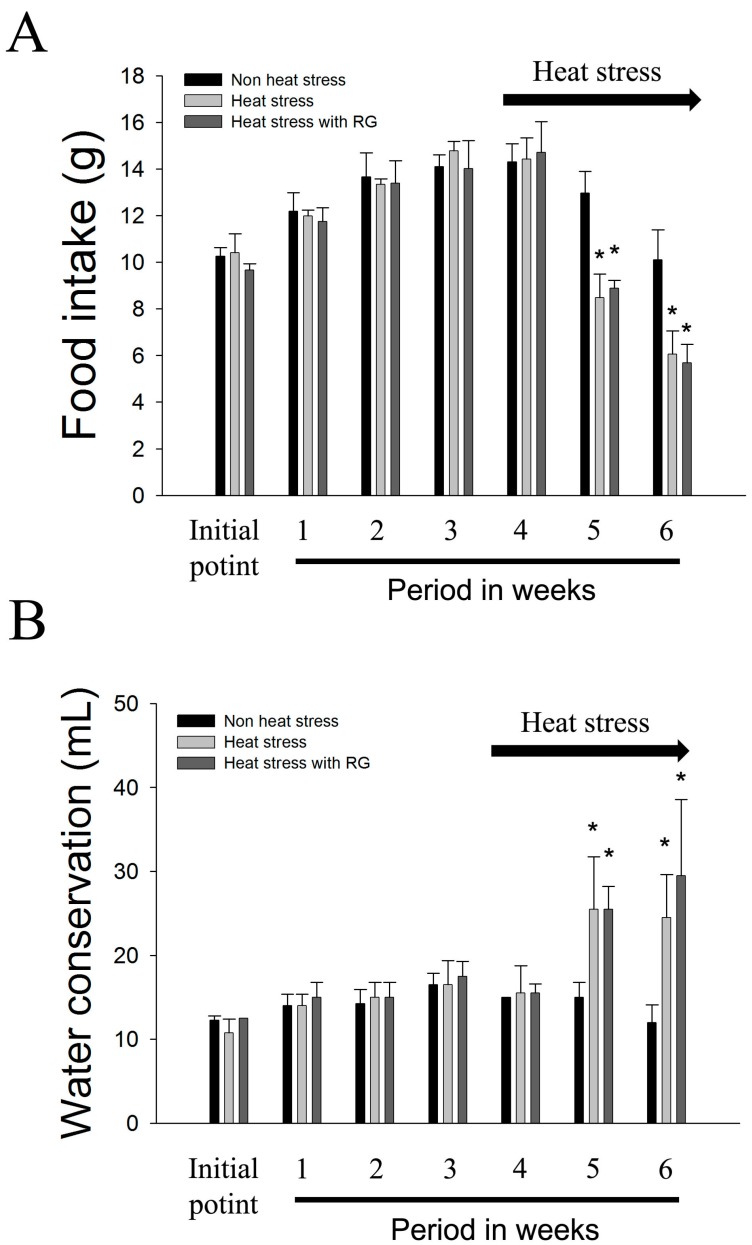
Mean of food intake and water conservation of SD rats housed under a normal environment for four weeks and with heat stress for two weeks. (**A**) Mean of food intake of SD rats; (**B**) Mean of water conservation of SD rats. The graph indicates control, heat stress, and heat stress with red ginseng extracts (RG) rats. The results are expressed as means ± standard deviation (*n* = 8). * *p* < 0.05 compared with heat stress animals.

Food intake and water consumption were measured in mg/kg/day at the start of treatment and at weekly intervals thereafter. Food intake and water conservation did not differ for all groups before heat exposure (Figure 3). There were significant decreases in food intake in both heat stress exposed groups. 

### 2.3. Red Ginseng Regulates Heat Stress Inducible Hepatic Gene Expression in Rat

RG extracts were prepared by traditional extraction method as shown in Figure 1 and were concentrated under vacuum at 60 °C and stored at 4 °C until use. In a previous study, we reported a number of hepatic gene expressions, such as for HSPA1, DEAF1 HMGCR, and FMO1, were changed by heat stress [9]. 

To determine the effect of RG on heat stress response gene expression profiles, we induced environmental heat stress with and without RG oral administration in SD rats. The semi-quantitative RT-PCR was performed on liver samples of rats after long term heat stress exposure. 

**Figure 4:**
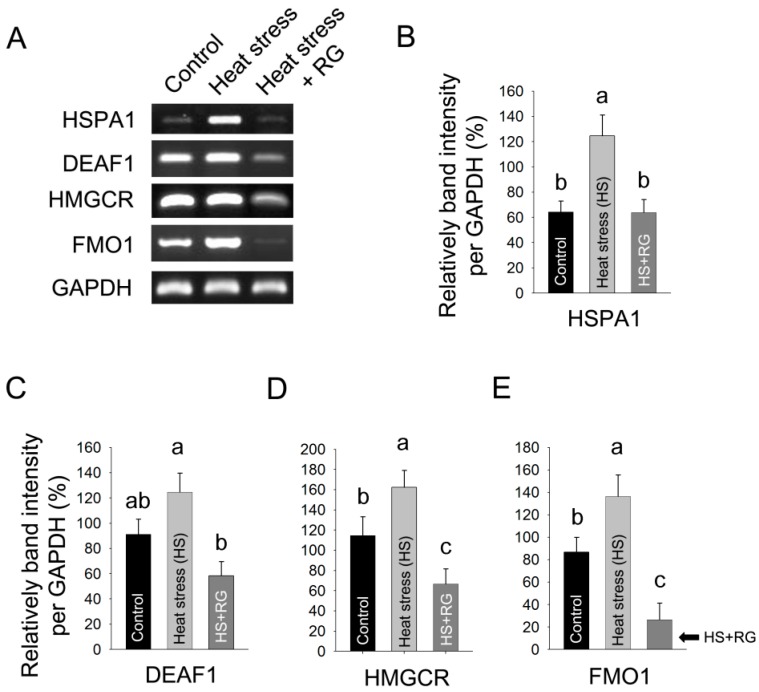
Semi-quantitative reverse transcription-PCR (RT-PCR). (**A**) Semi-quantitative RT-PCR was performed on the HSPA1, DEAF1, HMGCR, FMO1, and GAPDH mRNA in the control, heat stress and heat stress with orally administered red ginseng extracts (RG) groups; (**B**) The expression of HSPA1 was normalized by GAPDH; (**C**) The expression of DEAF1 was normalized by GAPDH; (**D**) The expression of HMGCR was normalized by GAPDH (**E**) The expression of FMO1 was normalized by GAPDH. The results are expressed as means ± standard deviation (*n* = 8). Values with different letters are significantly different, *p* < 0.05.

As shown in Figure 4, a representative RT-PCR for each of the four genes evaluated and the ratio of comparisons of the individual protein of the control group and of the group treated for the induced heat stress. For all comparisons, the mean ratios obtained from the gene expression analysis were very similar to those obtained from RT-PCR results. The heat exposure group increased HSPA1, DEAF1, HMGCR, and FMO1 mRNA expression, while the RG administrated group dramatically suppressed HSPA1, DEAF1, HMGCR, and FMO1. The results indicate RG have the capacity the prevention activity of heat stress via regulation of mRNA expression. The specific function of these genes is related to lipid metabolism and oxidative stress. 

### 2.4. Red Ginseng Suppresses Heat Environment Stress Induced Lipid Accumulation and Lipid Peroxidation Related Gene Expression

The lipid metabolism in the liver is regulated by a complex network involving lipogenesis, fatty acid oxidation, and lipid secretion [21]. The CCAAR/enhancer-binding protein (C/EBP) family members (C/EBPβ, C/EBPδ, and C/EBPα) play specific roles in regulating gene during the lipogenesis [22]. One of the major functions of C/EBPβ is to induce the peroxisome proliferator-activated receptor γ (PPAR γ) activity. Therefore, we decided to choose C/EBPβ to evaluate of hepatic lipid accumulation in rat subjected to heat stress. 

**Figure 5:**
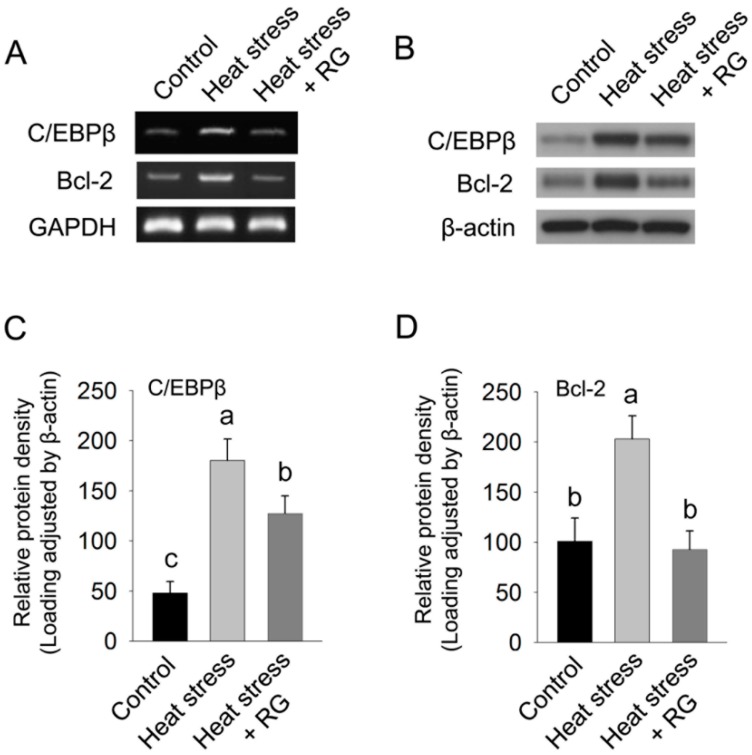
Red ginseng extracts (RG) suppressed the expression of C/EBPβ and Bcl-2 induced by heat stress. (**A**) The protein level of C/EBPβ, Bcl-2, and GAPDH was determined by semi-quantitative RT-PCR; (**B**) The protein level of C/EBPβ, Bcl-2, and GAPDH was determined by western blotting; (**C**) The protein expression of C/EBPβ was normalized by β-actin; (**D**) The protein expression of Bcl-2 was normalized by β-actin. The results are expressed as means ± standard deviation (*n* = 8). Values with different letters are significantly different, *p* < 0.05.

To address our hypothesis, we first evaluated the expression of C/EBPβ. As shown in Figure 5, the expression of C/EBPβ mRNA was induced in heat stress exposed group compared to the control group, whereas this induction was abrogated by RG administration. In addition, the protein expression of C/EBPβ in RG administrated groups also decreased by 29% compared with the heat stress group.

The consequence of hepatic lipid accumulation includes oxidative stress damage to DNA [23]. The Bcl-2 gene protects cell death against mitochondrial oxidative stress induced by heat stress [24]. Therefore, we evaluated the mRNA and protein expression of Bcl-2 in among control, heat stress, and the RG administrated group during heat stress. Bcl-2 gene is dramatically decreased to similar control levels when the rats were orally administered RG during environmental heat stress. 

**Figure 6:**
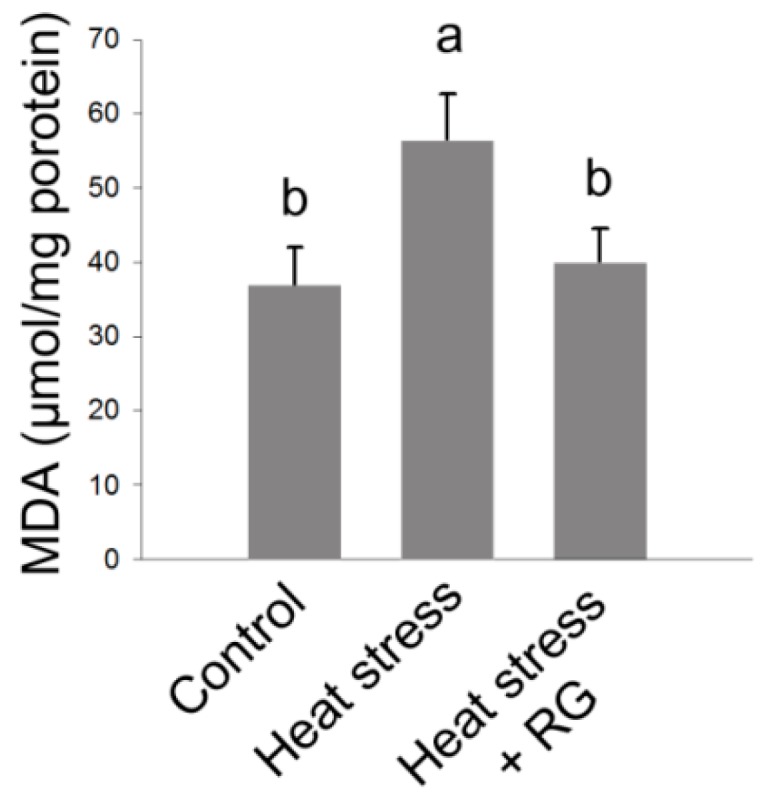
Effects of heat stress and Red ginseng extracts (RG) on lipid peroxidation in rat livers. The results are expressed as means ± standard deviation (*n* = 3). Values with different letters are significantly different, *p* < 0.05.

These data suggest that RG participates in lipid accumulation, but it also promotes inhibition of hepatic cell death by inhibiting Bcl-2 gene during environmental heat stress. Moreover, MDA contents were strongly suppressed in the RG oral administrated group compared to control as shown in Figure 6.

These results suggest that RG performs the liver protection activity due to inhibition of the lipid peroxidation and oxidative stress [25]. Thus, we investigated the effect of RG on hepatic oxidative stress in rats subjected to environmental heat stress. 

### 2.5. Effect of Red Ginseng on Hepatic Oxidative Stress in Environmental Heat Stress

To determine the effect of RG on hepatic oxidative stress in rat subjected to environmental heat stress, we evaluated the mRNA and protein expression of CuZnSOD and GPx, which play a crucial role in protecting cells from the harmful effects of ROS.

**Figure 7:**
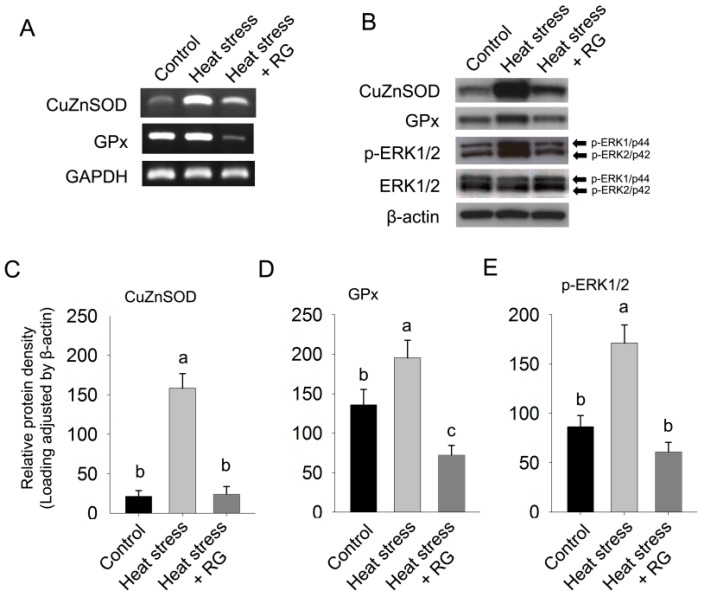
Oxidative stress related gene expression in the control, heat stress, and heat stress with orally administered Red ginseng extracts (RG) groups. (**A**) The protein level of CuZnSOD, GPx, and GAPDH was determined by semi-quantitative RT-PCR; (**B**) The protein level of CuZnSOD, GPx, p-ERK, ERK and β-actin was determined by western blotting; (**C**) The protein expression of CuZnSOD was normalized by β-actin; (**D**) The protein expression of GPx was normalized by β-actin; (**E**) The protein expression of p-ERK was normalized by β-actin. The results are expressed as means ± standard deviation (*n* = 8). Values with different letters are significantly different, *p* < 0.05.

As shown in Figure 7, a significant induction in CuZnSOD and GPx gene expression was observed in rats that received environmental heat stress as compared with rats that did not receive environmental heat stress. Whereas, CuZnSOD and GPx gene expression decreased to below control levels when the rats were administered RG orally during heat stress. The protein levels of CuZnSOD and GPx in the RG administrated group decreased to an activity significantly below the control levels. These results strongly suggest that RG inhibits oxidative damage due to environmental heat stress by regulating the CuZnSOD and GPx to oxidative stress. 

### 2.6. Discussion

The ultimate aim of this research is to determine the environmental heat stress therapeutic potential of RG in SD rat model. High temperatures have been demonstrated to adversely affect the physiology of cells, causing impairments in transcription, RNA processing, oxidative metabolism, and function [26]. 

There are a number of genes expression due to environmental heat stress that have been reported in rodents [27], and in mouse livers [9]. The expression levels of genes encode antioxidant enzymes have shown to be dramatically altered. In particular, environmental heat stress causes cellular damage and the abnormal accumulation of hepatic lipid through regulation of HSPA1, HMGCR, DEAF1, and FMO1 transcription factors. HSPA1 is expressed in response to the endogenous cellular stress and it against to cellular damage such as apoptosis [28,29]. HSPA1 interacts specifically with sterol and steroid ligands such as progesterone receptor [30,31]. The activation of progesterone receptor was shown to enhance the production of triglyceride in cells [32]. HMGCR is a key enzyme of lipid biosynthesis and is a sensitive physiological stress marker [33]. We and other groups showed that HSP70 and HMGCR can be appropriate biomarkers to assess the effect of environmental heat stress in rats and chickens [9,34]. The over-expression of DEAF1 enhances cell proliferation and disrupts tissue development during embryogenesis [35,36]. FMO1 is the most prominent isoform in adult hepatic liver and involved hepatic detoxification of a number of xenobiotic compounds [37,38,39]. FMO1 also stimulates microsomal omega oxidation to decrease fatty acid accumulation in the liver [40]. Thus, the proper regulation of the aforementioned gene in response to environmental heat stress is important to prevent the abnormal health conditions in humans. 

RG is the most popular medicinal herb in the Asian-Pacific region [41], and contains higher contents of bioactive ingredients, such as the acidic polysaccharide, saponin, and ginsenoside, than other forms of manufactured ginseng products in general [42,43]. There are two major types of ginsenoside in RG. One group is characterized by protopanaxadiol such as Rb1, Rb2, Rb, and Rd. The other group is characterized by protopanaxatriol such as Rg1, Re, Rf, and Rg2. It has been shown that ginsenoside prevents multiple types of stress [44,45,46]. However, the causal relation between environmental heat stress and RG in *in vivo* is poorly understood.

Here, we show that RG suppresses environmental heat stress induced genetic instability *in vivo*. Variable effects of RG on body weight, food intake, and water conservation have been observed in rodent studies [47,48,49]. In this study, the rats exposed to heat stress exhibited a much smaller body weight change when compared with the control, consistent with our previous studies [9,10]. The rats that received a treatment of orally administered RG during heat stress had no change compared to heat stress group. Similar to this result, heat stress with or without RG groups decreased several organ weights.

In addition, our results showed that these mRNA expressions were dramatically decreased in the RG administered group under environmental stress compared with the environmental heat stress group with absence of RG. Previous reports have shown that C/EBPβ deletion attenuates lipid accumulation [50]. Upon lipid accumulation, C/EBPβ is transiently induced, followed by induction of PPARγ [22]. A constituent of RG initiates the repression of lipid accumulation through the inhibition of PPARγ [51]. Our data consistently showed that C/EBPβ was dramatically suppressed in the RG administered group during heat stress compared to the unadministered heat stress group. Moreover, heat stress induces lipid peroxidation because of the generation of free radicals, as indicated by the MDA contents [25], has been demonstrated previously. We examined to determine whether RG could suppress lipid peroxidation due to induced heat stress in rats. We observed that MDA concentration was significantly increased in the heat stress group, whereas RG repressed the induction of MDA concentration due to heat stress. The lipid accumulation sensitizes the liver to events that lead to inflammation and is associated with serves liver damage [52,53]. 

The Bcl-2 protein expression is associated with the presence of inflammation and lipid peroxidation [54]. We found that RG suppressed heat stress induced Bcl-2 expression in both mRNA and protein. These results indicate RG prevents not only an induction of lipid accumulation, but also inhibits lipid peroxidation by regulating a change in the expression of transcripts related to lipid peroxidation and suppresses hepatocyte apoptosis caused by heat stress. 

The induction of CuZnSOD and GPx is more likely to occur as a consequence of a cellular adaptive survival response to oxidative stress caused by environmental heat stress. RG depressed the expression of endogenous anti-oxidant enzymes, protopanaxadiol induces apoptosis through endoplasmic recticulum stress in human hepatic isolated primary cell line [55], whereas the other groups have shown that protopanaxadiol type of ginsenoside has a protective effect of chronic hepatotoxicity in mice [56]. In addition, protopanaxatriol also can suppress chemical induced apoptosis and cell death by modulating intracellular stress status [57,58,59]. These data strongly support our major observation. Thus, we can speculate that RG includes both types (protopanaxdiol and protopanaxatriol) of ginsenoside such as Rb1 and Rg1 and subsequently inhibits heat environmental stress due to repression of oxidative stress in mice.

## 3. Experimental Section

### 3.1. Materials

Red ginseng was purchased Nonghyup central institute (Seoul, Korea). The sodium carbonate, gallic acid, β-d-galacturonic acid, carbazole ethanol, sulphuric acid were purchased from Sigma (St. Louis, MO, USA). Phosphate buffered saline (PBS) was acquired from Gibco (Gaithersburg, MD, USA). The RNA*later* and RNeasy Mini Kits were purchased from Ambion (Austin, TX, USA) and Qiagen (Hilden, Germany), respectively. The First-Strand cDNA Synthesis Kit and Taq DNA polymerase were obtained from Invitrogen (Carlsbad, CA, USA) and Solgent (Daegeon, Korea), respectively. Primers were supplied by Bioneer (Daejon, Korea).

### 3.2. Animal Husbandry and Maintenance

A total of 16 specific pathogen-free, four-week-old male Sprague Dawley (SD) rats weighing between 218.9 and 238 g were purchased from Japan SLC, Inc. (Shizuoka, Japan) and used after a week of quarantine and acclimation. Rats were housed in a light-controlled room (light 07:00~19:00) maintained between 23 to 26 °C with humidity of 50% to 55% and light intensity of 100 to 200 Lux. The animals were allowed sterilized tap water and commercial rodent chow (AIN 76A, Diets, Philadelphia, PA, USA) ad libitum. The experiment was performed in accordance with the guidelines for animal experimentation provided by the Faculty of Agriculture, CHA University (Seongnam, Gyonggi, Korea). After a one-week adaptation period, the rats were assigned to three groups (*n* = 8) using a randomized complete block design. The study was approved by the CHA University Institutional Animal Care and Use Committee (IACUC), and the care of all study animals was in accordance with IACUC guidelines.

### 3.3. Heat Environmental Stress Study Design

One group was housed at room temperature and provided with a basal diet for six weeks. The other group was provided with a basal diet for four weeks and then subjected to heat stress (temperature, 37~38 °C) while receiving a basal diet for two weeks. After the treatment period ended, the rat liver, spleen, and kidney were excised and weighed; the liver was cut into small pieces, which were frozen in RNA*later* (Ambion, Austin, TX, USA) and stored at −80 °C until analysis.

### 3.4. Preparation of Red Ginseng Extracts 

The red ginseng was dried and extracted with deionized water at 20 times volume; three extractions were carried out for 3 h at 80 °C. The resulting extracts were filtered, concentrated under vacuum at 60 °C and stored at 4 °C until use.

### 3.5. Measurement of Total Phenolic Content in Red Ginseng 

The total phenolic content was determined according to the Folin–Denis Method [60]. The sample solution (1 mL) was placed in a test tube with distilled water (7 mL) and Folin-Ciocalteu reagent (0.5 mL), saturated with sodium carbonate solution (1 mL), and allowed to stand for 30 min. The absorbance was measured at 715 nm. The total phenolic content was calculated as gallic acid equivalents (GAE).

### 3.6. Determination of Acidic Polysaccharide in Red Ginseng 

The amount of acidic polysaccharides was analyzed by the carbazole-sulphuric acid method [61], using β-d-galacturonic acid as a standard. Fermentation liquor (0.5 mL) was mixed with 0.25 mL of 0.1% carbazole ethanol and 3 mL of concentrated sulphuric acid prior to incubation at 80 °C for 5 min. After allowing the solution to cool for 15 min at room temperature, the acidic polysaccharide is quantitatively analyzed by spectrophotometric determination at 525 nm.

### 3.7. RNA Isolation and Semi-quantitative RT-PCR

Total RNA was extracted from the Rat subjected liver using the Qiagen RNeasy Kit (Qiagen, Valencia, CA, USA) according to the manufacturer’s recommended procedures. The quality of total RNA was estimated based on the integrity of 28S and 18S rRNA. rRNA was separated using 1% agarose gel electrophoresis. Good RNA quality was demonstrated by the fact that the 28S rRNA band had twice the intensity of the 18S rRNA band; no significant smearing was observed for the rRNA bands. Samples of total RNA from the three rats exposed to the same dose of heat stress were pooled for subsequent use in the GeneChip analysis. Total RNA samples with an OD_260_/OD_280_ ratio higher than 2.0 were used for semi-quantitative RT-PCR. One microgram of total RNA was used to produce cDNA using an RT-PCR system. The sequence of the oligonucleotide primers were as Table 3. PCR products were run on 1.5% agarose gels, stained with ethidium bromide and photographed. Densitometric analysis was performed using ImageJ software (National Institute of Health, Bethesda, MD, USA).

**Table 3:** PCR primers used for semi-quantitative RT-PCR.

Gene	Sense Primer, 5′→3′	Antisense Primer, 5′→3′
GAPDH	TGATGACATCAAGAAGGTGG	TTTCTTACTCCTTGGAGGCC
HMGCR	GAGGCGCAACTGGAAACT	GCGGACGTCTGTGTAGAAGA
DEAF1	AGAAGCCATCACAGGCTTAG	AGAAAGAGCACCGTGATAGG-
FMO1	TGTCAAGGGAAGCAAAGC	CCTGAATCAAAGACTCGGC
HSPA1	CTTACCTGGGCCAGAAGGT	ATGGTCAGGATGGACACGT
CuZnSOD	GTTCCGAGGCCGCCGCGCGT	GTCCCCATATTGATGGAC
GPx	CTCTCCGCGGTGGCACAGT	CCACCACCGGGTCGGACATAC

### 3.8. Preparation of Liver Homogenate for Malondiadehyde

Rat liver were homogenized (Daihan, Seoul, Korea) in ice-cold 0.1 M PBS (pH 7.4) solution. Homogenates were centrifuged (Hanil, Seoul, Korea) at 10,000 × *g* for 1 h at 4 °C to remove cell debris, nuclei and mitochondria. Resulting supernatants were used for MDA measurements. MDA levels were determined by monitoring thiobarbituric acid (TBA)-reactive substances according to the method of Ohkawa *et al*. [62]. The samples read in a spectrophotometric plate reader at 540 nm.

### 3.9. Western Blot Analysis

Tissue was collected in lysis buffer (50 mM HEPES, 137 mM NaCl, 1 mM MgCl_2_, 1 mM CaCl_2_, 10 mM Na_2_P_2_O_7_, 10 mM NaF, 2 mM ethylenediaminetetraacetic acid (EDTA), 10% glycerol, 1% Igepal CA-630, 2 mM vanadate, 10 mg/mL leupeptin, 10 mg/mL aprotinin and 2 mM, pH 7.4, phenylmethylsulphonyl fluoride) [63]. Cell lysates were clarified by centrifugation at 12,000 *g* for 20 min at 4 °C; the amount of protein in the supernatants was determined using the Bradford Protein Assay (Bio-Rad Laboratories, Richmond, CA, USA). Proteins were directly solubilized in Laemmli sample buffer. Equal amounts of proteins were separated by SDS-polyacrylamide gel electrophoresis and transferred to Immobolin-P membranes. Membranes were blocked overnight at 4 °C and incubated with the indicated antibody for 2 h at room temperature. Specifically bound primary antibodies were detected using a peroxidase-coupled secondary antibody and enhanced chemiluminescence system (Amersham Biosciences, Buckinghamshire, UK).

### 3.10. Statistical Analysis 

All measurements were given as means ± SD. The results were statistically analyzed by Student’s t-test or one-way ANOVA and Duncan’s multiple range tests. Statistical significance was accepted at a level of *p* < 0.05 (SAS Inst., Inc., Cary, NC, USA).

## 4. Conclusions

In conclusion, we showed the environmental heat stress abnormally caused the over-expression of hepatic genes such as HSPA1, DEAF1, HMGCR, and FMO1, which contribute to regulate the lipid peroxidation, hepatic lipid accumulation, and oxidative stress, while RG dramatically decreased the expression of HSPA1, DEAF1, HMGCR, and FMO1 genes in rats subjected to environmental heat stress. Our result was consistent and showed C/EBPβ, initial stage of lipid accumulation regulatory gene, and Bcl-2, cellular damage associated gene, were downregulated in rats subjected to environmental heat stress with the presence of RG compared to heat stress group. In addition, the activity of CuZnSOD, GPx and the contents of MDA, the parameter of lipid peroxidation, in livers of rats subjected environmental heat stress were increased, however, RG attenuated the rise in the activity of CuZnSOD, GPx and the contents of MDA, in rats subjected environmental heat stress. Moreover, the composition analysis of RG revealed that RG included the large amounts of ginsenoside Rg1, Rg3, Rb1, and Rb2. Therefore, RG or the bioactive compounds of RG may be new therapeutic material to prevent environmental heat stress through regulation of the oxidative stress and hepatic cellular damage in humans.
